# Cytoplasmic Her2/neu Immunohistochemical Staining in Breast Cancer; From a Molecular Point of View

**DOI:** 10.30699/ijp.2019.76630.1732

**Published:** 2019-07-01

**Authors:** Akbar Safaei, Ahmad Monabati, Maral Mokhtari, Mehdi Montazer

**Affiliations:** 1 Department of Hematopathology, Molecular Pathology and Cytogenetics, Shiraz University of Medical Sciences, Shiraz, Iran

**Dear Editor,**

The most widely used guideline for the breast cancer biomarker assessment and reporting (the 2013 ASCO/CAP guideline) does not state the unusual occurrence of cytoplasmic Her2/neu staining (1, 2). 

**Figure 1:**
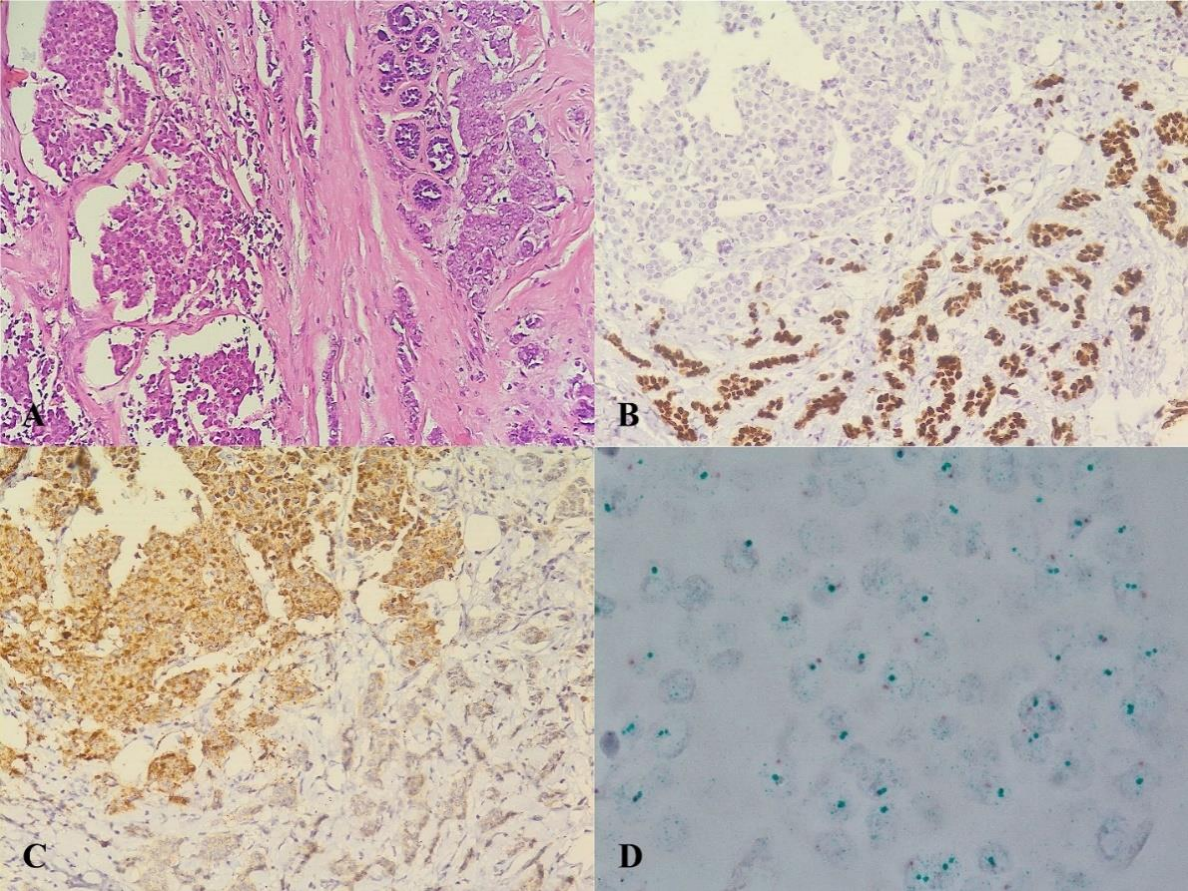
A: Two distinct tumor cell populations. Neuroendocrine-like component (NEL) (left) and usual ductal component (UAC) (right) (original magnification, 20X; H&E stain). B and C: PgR and Her2/neu immunohistochemical stainings, respectively. NEL shows cytoplasmic Her2 and completely lacks PgR (NEL at upper left, UAC at lower right) (original magnification, 20X). D: Dual color chromogenic in situ hybridization showing no amplification of Her2/neu gene in NEL

We recently encountered a T2N1Mx ductal adeno-carcinoma which consisted of two dissimilar tumor cell populations. The more prominent population (75% of tumor cells) was made up of sheets of neuroendocrine-like cells (NEL) and the other tumor cell population had a usual adenocarcinomatous histomorphology (UAC) (Fig. 1A). The NEL was ER+ (clone 073), PgR-(clone 636), 40% ki67 with distinct dot-like cytoplasmic Her2 staining (clone CB11) which is considered as negative regarding the current guidelines. The UAC was ER+, PgR+, 20% ki67, and Her2 negative (Fig. 1A-C). Moreover, NEL did not react with either chromogranin or synaptophysin, but it expressed neuron-specific enolase (NSE). Dual color Her2/neu chromogenic in situ hybridization probes (chromogenic ISH) established that both components were not amplified for this oncoprotein gene (Fig. 1D). 

Few papers have previously noted cytoplasmic Her2 staining. They have found, as we did, that this pattern was not associated with the Her2 gene amplification or high Her2 mRNA levels, and some of them therefore attributed it to the artifactual staining by the routinely employed CB11 Her2 clone (1). As we know, PgR and Her2 are in reverse association with each other, and since the areas with cytoplasmic Her2 completely lack PgR in our case, we prefer to consider that CB11 cytoplasmic staining means the true presence of Her2 oncoprotein in cytoplasm and is not a nonspecific reaction ensued from some kind of cross-reaction or technical error. In addition, the intracytoplasmic isotype of Her2 has been previously described which has a different molecular weight from the membraneous one (3). 

Interestingly, Horiguchi et al. has shown that intracellular localization of Her2 molecule is associated with the neuroendocrine features. Our case also expressed one neuroendocrine marker, NSE, although more specific neuroendocrine markers were absent (4). 

The prognostic effect and targetability of cytoplasmic Her2 is remained unknown in breast cancer because the related studies belong to the pre-trastuzumab years and the ASCO/CAP guideline does not endorse prescribing trastuzumab for the non-membraneous Her2 reactions. Unfortunately, this guideline does not express either the expert ideas about the gene expressivity and Her2 mRNA levels which may cause high Her2 expression without gene amplification (1, 5). 

In our opinion, the exact nature and consequences of cytoplasmic Her2 staining is still a subject to more discussions and investigations. We recommend considering this phenomenon in the next ASCO/CAP guideline.
